# miR-17/20 sensitization of breast cancer cells to chemotherapy-induced apoptosis requires *Akt1*

**DOI:** 10.18632/oncotarget.1804

**Published:** 2014-03-04

**Authors:** Zuoren Yu, Zengguang Xu, Gabriele DiSante, Jennifer Wright, Min Wang, Yuan Li, Qian Zhao, Tao Ren, Xiaoming Ju, Ellen Gutman, Guangxue Wang, Sankar Addya, Tieyan Li, Zhendong Xiang, Chenguang Wang, Xiongfei yang, Xiaolai Yang, Richard Pestell

**Affiliations:** ^1^ Research Center for Translational Medicine, Key Laboratory of Arrhythmias of the Ministry of Education, East Hospital, Tongji University School of Medicine, Shanghai, China; ^2^ Departments of Cancer Biology, Kimmel Cancer Center, Thomas Jefferson University, Philadelphia PA; ^3^ People's Hospital of Gansu Province, Lanzhou, China

**Keywords:** miR-17/20, breast cancer, AKT, apoptosis

## Abstract

The serine threonine kinase Akt1 has been implicated in the control of cellular metabolism, survival and growth. Herein, disruption of the ubiquitously expressed member of the Akt family of genes, Akt1, in the mouse, demonstrates a requirement for Akt1 in miRNA-mediated cellular apoptosis. The miR-17/20 cluster is known to inhibit breast cancer cellular proliferation through G1/S cell cycle arrest via binding to the cyclin D1 3'UTR. Here we show that miR-17/20 overexpression sensitizes cells to apoptosis induced by either Doxorubicin or UV irradiation in MCF-7 cells via Akt1. miR-17/20 mediates apoptosis via increased p53 expression which promotes Akt degradation. Akt1 *−/−* mammary epithelial cells which express Akt2 and Akt3 demonstrated increased apoptosis to DNA damaging agents. Akt1 deficiency abolished the miR-17/20-mediated apoptosis. These results demonstrated a novel pathway through which miR17/20 regulate p53 and Akt controlling breast cancer cell apoptosis.

## INTRODUCTION

The cell survival oncoprotein *Akt1*, also known as protein kinase B (PKB), is frequently hyperactivated in human cancers. *Akt1* is recruited to the plasma membrane in the presence of phosphoinositide triphosphate (PI-3,4,5-P). *Akt1* plays a central role in the ability of external signals to promote cell survival by preventing cytochrome c release from mitochondria (1-3) and maintaining mitochondrial membrane integrity by increasing hexokinase (HK) association with mitochondria (4). In mammalian cells, activating growth factors and oncogenes stimulate *Akt1* kinase activity to promote anti-apoptotic signaling (4). Three separate genes with high sequence identity encode the major isoforms of *Akt1/PKB* (*Akt1/PKBα, Akt2/PKBβ, Akt3/PKBγ*). Substrate specificity of the *Akt1* isoforms is similar, although *Akt1* is the predominant isoform expressed in most tissues. Constitutive activation of *Akt1* kinase occurs in human cancer through deletion and mutation of the tumor suppressor gene *PTEN*, the phosphatase that negatively regulates *Akt1*, through amplification of the *Akt1* genes, or through amplification of the catalytic subunit of PI3 kinase (5-7). The pro-proliferative and prosurvival effects induced by *Akt1* kinase are conducted through regulation of caspase 9, I B kinase, Bad, and induction of the GSK3/cyclin D1 signaling pathways (reviewed in: (8, 9)).

ErbB2/ErbB3 receptor activation, which occurs frequently in breast cancer, induces PI3K and *Akt1* kinase activity (10, 11). The *ErbB2* oncogene is amplified in up to 30% of human breast cancers and is associated with poor patient prognosis in response to chemotherapeutic agents. ErbB2 induces *Akt1* activity, cellular growth and therapeutic resistance (12). The activation of ErbB2 is an early event in human breast cancer with ErbB2 overexpressed in up to 80% of primary ductal carcinoma *in situ* lesions (13). MicroRNAs (miRNAs) are 21-22 nucleotide molecules that regulate the stability or translational efficiency of targeted messenger RNAs. Derived from nuclear precursor RNAs, initial processing occurs by the inter-nuclease Drosha to release pre-miRNA of 60-70 nucleotides in length from pri-miRNA. Subsequent transport to the cytoplasm by exportin-5 results in processing by the inter-nuclease Dicer to generate the ~22 nucleotide mature miRNA (14-16).The base pairing interactions between miRNAs and their target mRNAs, often within the 3' untranslated region (3'UTR) of target genes, results in the degradation of target mRNAs (17, 18) or inhibition of their translation (19). To date more than 2,000 miRNAs have been identified or predicted in humans (miRBase Sequence Database Version 20.0 released in Jun. 2013). It has been proposed that as each vertebrate miRNA may bind to as many as 200 gene targets, miRNAs potentially control the expression of about one-third of human mRNAs (20).

Several independent lines of evidence support a role for miRNAs in human cancer (21-25). miRNA encoding genes are frequently located at fragile sites, and in minimal regions of loss of heterozygosity, minimal regions of amplification, and in common breakpoint regions involved in cancers (26). Aberrant expression of miRNAs or mutations of miRNA genes have been described in many types of tumors. Let-7 abundance is reduced in several cancers including lung cancer (27), and let-7 was reported to regulate tumor growth by targeting the *ras* gene (28). miR-15a and miR16-1 were deleted and/or down-regulated in ~ 70% of patients with chronic lymphocytic leukemia (29). miR-15a/16-1 induced apoptosis by inhibiting BCL-2 (30). The miR-34 family is an important component of the p53 tumor suppressor network (25). The human miR-17/20 cluster's genomic location, chromosome 13q31, correlates with loss of heterozygosity in a number of different cancers including breast cancer (31, 32). The expression and function of miRNA varies by cell type. The miR-17/20 cluster functions as a tumor suppressor in human breast cancer by decreasing *AIB1* and *cyclin D1* expression (33, 34). In contrast, in both lung cancer and lymphomas, expression of this miRNA cluster was increased, enhancing cell growth (22, 35). The onset and progression of tumorigenesis involves evasion of apoptotic signals, sustained cellular proliferation and the ability to promote tumor neoangiogenesis. As the same miRNA performs different functions through distinct pathways dependent on the tissue or cell type, it is important to understand the mechanisms by which miRNA regulates the cellular apoptosis and thereby tumorigenesis.

## RESULTS

### miR-17/20 sensitized stress signal-induced apoptosis in breast cancer cells

**Figure 1:**
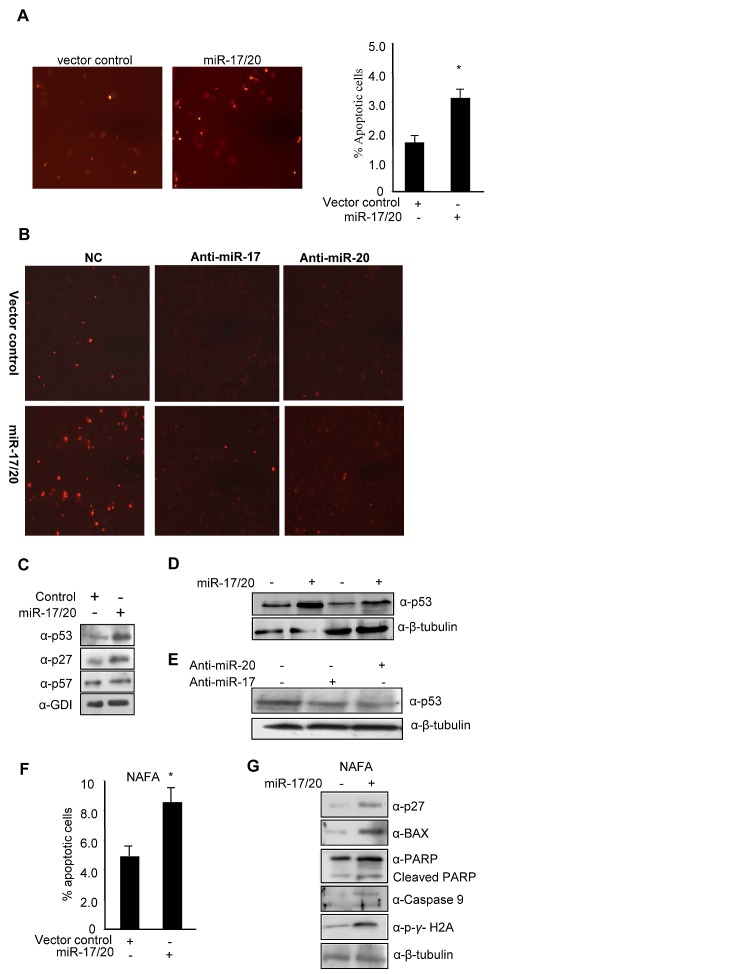
miR-17/20 sensitizes doxorubicin-induced apoptosis, and increase p53 expression in breast cancer cells A, Tunnel Assay on miR-17/20 or control transduced MCF-7 cells after treatment with doxorubicin at 0.05uM for 24h. B, Tunnel Assays on anti-miR-17-, anti-miR-20a- and negative control-transfected MCF-7 cells after treatment with doxorubicin (0.05μM) for 24h. C, Western blots showing the increased p53 and p27KIP1 in miR-17/20 transduced MCF-7 cells. GDI served as loading control. D, Western blots showing the increased p53 expression in miR-17/20 transduced MCF-7 cells in the presence (lane 1 and 2) or absence (lane 3 and 4) of doxorubicin treatment. E, Western blot showing decreased p53 in anti-miR-17 and anti-miR-20a- transduced MCF-7 cells. F, miR-17/20 sensitizes NAFA cells to doxorubicin-induced apoptosis. G, Western blot showing increased p27KIP1 BAX, p-γ/H2A, PARP and Caspase 9 in miR-17/20 transduced NAFA cells. β-Tubulin served as loading control.

Our previous studies demonstrated the suppression of cellular proliferation in human breast cancer cells by miR-17/20. In order to determine the potential role of miR-17/20 in regulating breast cancer cell apoptosis, MCF-7 cells were transduced with a retrovirus encoding the miR-17/20 cluster. The cells were treated either with 0.05 μM doxorubicin for 24 hours or with UV radiation and then analyzed for apoptosis by TUNEL assay. Apoptotic cells were increased in miR-17/20 overexpressing MCF-7 cells compared to control cells. Thus, miR-17/20 increased MCF-7 sensitivity to DNA damage inducing agents after doxorubicin or UV radiation (Fig. 1A and Supplemental Fig. S1).

In order to corroborate the effects of miR-17/20 on apoptosis, anti-miR-17 and anti-miR-20a were applied to block the function of endogenous miR-17 and miR-20a. In MCF-7 cells, doxorubicin-induced cellular apoptosis, assessed by TUNEL analysis, was abolished by anti-miR treatment in miR-17/20 transduced MCF-7 cells (Fig. 1B).

In order to identify the mechanisms by which miR-17/20 regulates cellular apoptosis, analysis was conducted of apoptosis-regulating pathways and target genes (p53, p57, Cyto C, Caspase 3, Caspase 9, Bax, and PARP). The expression of p53 and p27^KIP1^ increased in miR-17/20 transduced MCF-7 cells. p57 expression was unchanged by miR-17/20 treatment (Fig. 1C). Doxorubicin treatment of MCF-7 cells enhanced miR-17/20 induction of p53 (Fig. 1D). Consistent with the observation, transfection of MCF-7 cells with anti-miR-17 or anti-miR-20a decreased p53 expression (Fig. 1E).

**Figure 2:**
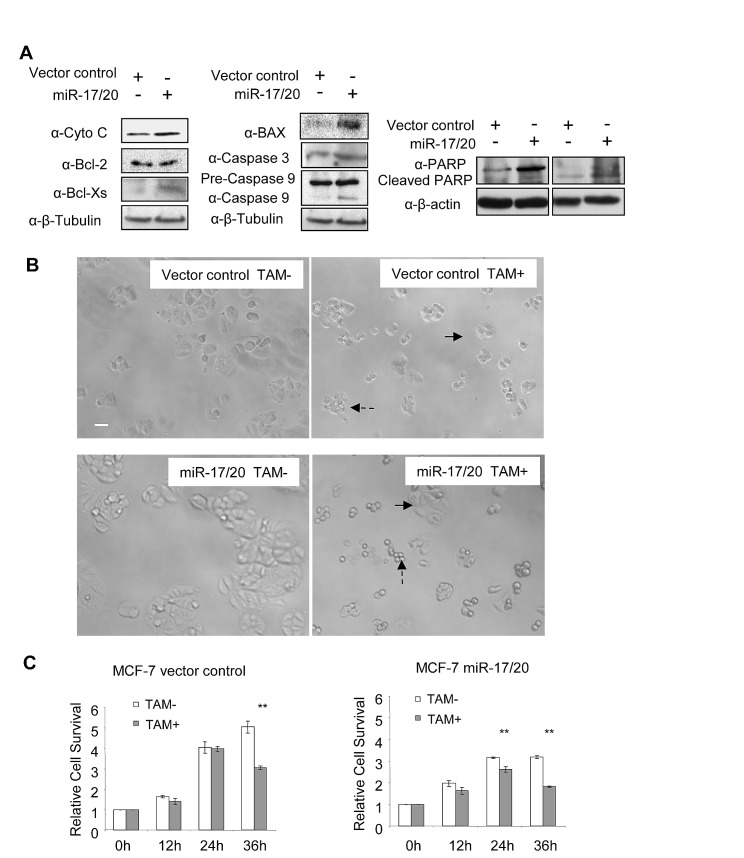
miR-17/20 increases tamoxifen sensitivity of MCF-7 cells A, Western blot showing the regulation of apoptosis pathway-related genes in miR-17/20 transduced MCF-7 cells. B, Phase contrast images of miR-17/20 or control transduced MCF-7 cells after 36h treatment (15uM tamoxifen). Survived cells are indicated with solid arrow, apoptotic cells are indicated with dashed arrow. C, MTT assays measuring the relative survival rate of MCF-7 cells after treatment with tamoxifen (15uM) for the indicated times. The data are mean +SEM (n=5), **p<0.01.

The effect of miR-17/20 was next assessed in the NAFA cell line which was derived from an ErbB2-induced mouse mammary tumor. miR-17/20 transduction increased the sensitivity of NAFA cells to doxorubicin-induced apoptosis as assessed by TUNEL staining (Fig. 1F). The induction of apoptosis in miR-17/20 transduced NAFA cells was associated with induction of p27, Bax, p-γ-H2A, caspase 9 and cleaved PARP (Fig. 1G and Supplementary Figure S2). Similarly, miR-17/20 induced apoptosis in MCF-7 cells was associated with induction of Bax, Cyto C, Bcl-xs, caspase 3 and cleavage of caspase 9 and PARP (Fig. 2A).

### miR-17/20 increased MCF-7 cell sensitivity to tamoxifen

The anti-estrogen tamoxifen is the most commonly used treatment for patients with estrogen-receptor α (ER)-positive breast cancer. Tamoxifen resistance occurs in ERα+ breast cancer cells including MCF-7. miR-17/20 transduced MCF-7 cells and control were treated with 15 uM tamoxifen up to 36 hours. The relative cell survival was determined using the MTT assay. As shown in Figure 2B, miR-17/20 overexpression attenuated relative cell survival in the presence of tamoxifen (Fig. 2B, C). MCF-7 control cells showed sensitivity to 15 uM tamoxifen after 36h treatment while miR-17/20 transduced MCF-7 cells showed sensitivity after 24 hours treatment when compared to cells without tamoxifen treatment (Fig. 2C). At both 24h and 35h timepoints, control MCF-7 cells showed more resistance to tamoxifen than miR-17/20 transduced cells (Fig. 2C).

### miR-17/20 attenuated doxorubicin resistance in MCF-7 cell

**Figure 3:**
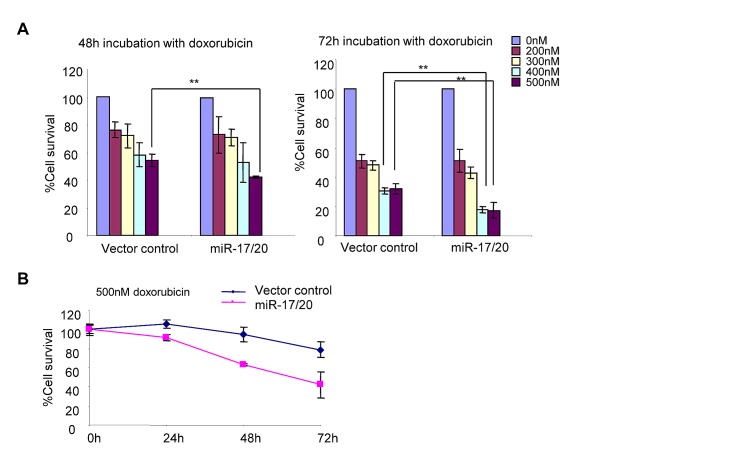
miR-17/20 increases the sensitivity of MCF-7 cells to doxorubicin A, Cell survival of miR-17/20 or control transduced MCF-7 cells after treatment with doxorubicin at indicated concentrations for 48h and 72h. B, Cell survival curves of miR-17/20 and control transduced MCF-7 treated with 0.5 μM doxorubicin for 0, 24, 48, and 72 hours. The data represent mean + SEM (n=5).

MCF-7 cells were treated with different concentrations (0-500 nM) of doxorubicin for 48h and 72h, followed by quantitative analysis of cell survival. Compared to control cells, miR-17/20 transduced MCF-7 showed increased sensitivity to 400 nM and 500 nM doxorubicin. The reduction in cell survival of miR-17/20 transduced cells was most pronounced (~20% vs. ~35%) after 72h of treatment (Fig. 3A). miR-17/20 overexpression decreased the IC_50_ of MCF-7 cells to doxorubicin (48h treatment). The MCF-7 cell growth curve with 500 nM doxorubicin treatment demonstrated significantly enhanced sensitivity to doxorubicin to doxorubicin after 24h of treatment (Fig. 3B).

### Akt1 is required for miR-17/20 sensitization of breast tumor cells to doxorubicin-induced apoptosis

**Figure 4:**
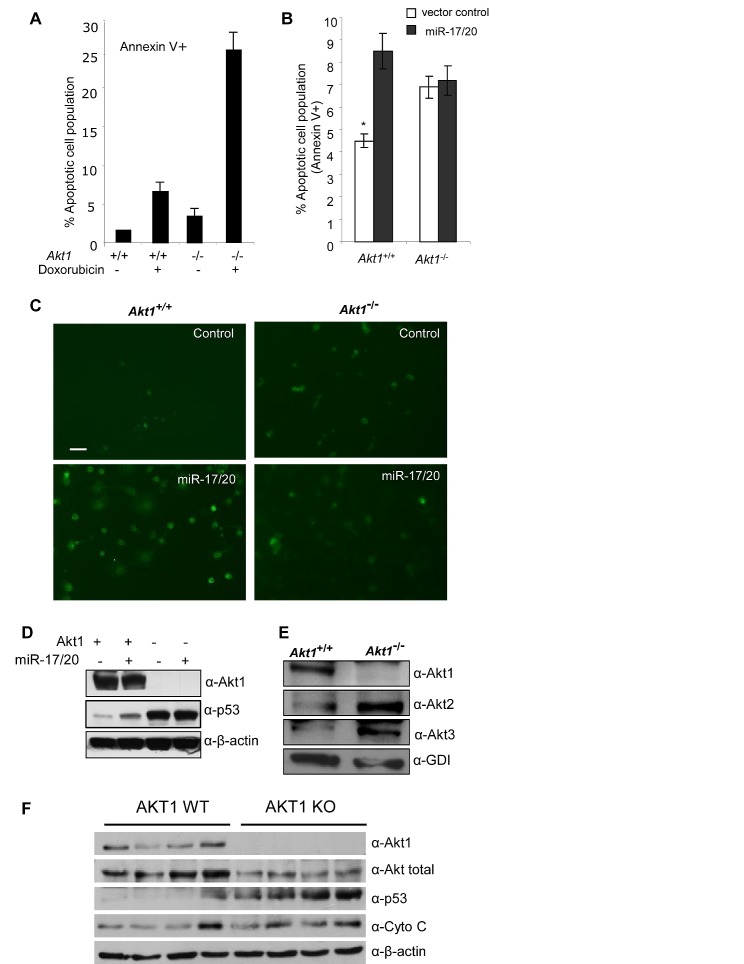
Akt1 is required for miR-17/20 sensitization of breast tumor cells to doxorubicin-induced apoptosis A, Annexin V staining showing the increased apoptosis in Akt1−/− murine ErbB2 breast tumor cells treated with doxorubicin at 0.5μM for 24h. B, Annexin V staining as a marker of apoptosis in miR-17/20 transduced Akt1+/+ or Akt1−/− murine ErbB2 breast tumor cells, **p<0.01. C, Tunnel assays for apoptosis in Akt1-/ - vs. Akt1+/+ murine ErbB2 breast tumor cells. D, Western blot of miR-17/20 transduced Akt1−/− and Akt+/+ murine ErbB2 breast tumor cells for p53. β-Tubulin is a loading control. E, Western blot of Akt1, Akt2 and Akt3 expression in Akt1−/− murine ErbB2 mammary tumor cells. GDI served as loading control. F: Western blot of Akt1, total Akt, Cyto C and p53 expression in multiple WT and Akt1−/− murine ErbB2 mammary tumors.

*Akt* and p53 both are important regulators of stress signal-induced cellular apoptosis (36). miR-17/20 sensitized the ErbB2-induced NAFA cell line to apoptosis. *Akt1* is required for the progression of ErbB2-induced breast tumorigenesis in transgenic mice (37). We investigated whether *Akt1* is involved in the miR-17/20 sensitized cellular apoptosis. We therefore derived breast tumor cell lines from MMTV-ErbB2/*Akt1^−/−^* and MMTV-ErbB2/*Akt1^+/+^* litter mate control mice and thereby assessed the role of *Akt1* in miR-17/20 mediated apoptosis. The mammary tumor cell lines were transduced with either miR-17/20 or control. Apoptosis assays indicated more apoptotic cells in *Akt1^−/−^* tumor cells than that in *Akt1^+/+^* cells after doxorubicin treatment (Fig. 4A). In addition to doxorubicin, puromycin was applied to *Akt1^+/+^* and *Akt1^−/−^* breast tumor cells to analyze the induced cellular apoptosis. As shown in supplementary Figure S3, *Akt1^−/−^* cells were more sensitive to puromycin than *Akt1^+/+^* cells at the indicated concentrations.

**Figure 5:**
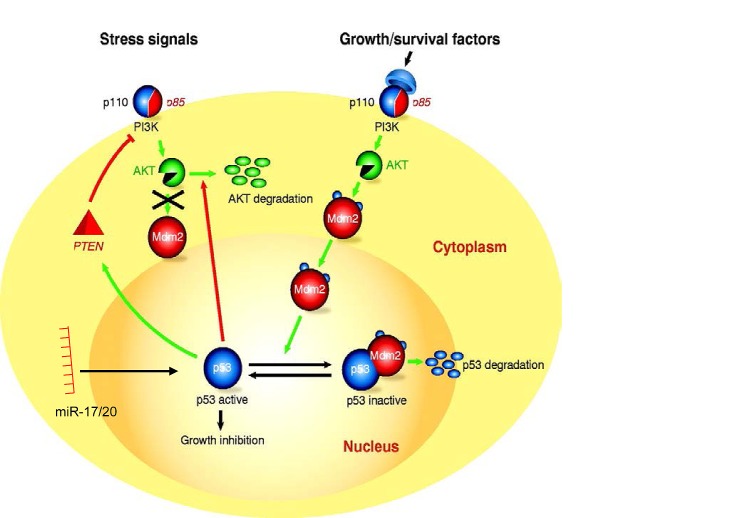
Schematic representation of the molecular mechanisms by which miR-17/20 and Akt1 regulate p53 abundance and thereby apoptosis.

miR-17/20 transduction was able to sensitize the *Akt1^+/+^* cells to doxorubicin treatment, but not *Akt1^−/−^* cells (Fig. 4B, C). Western analysis showed that miR-17/20 overexpression promoted p53 expression in *Akt1^+/+^* cells rather than *Akt1^−/−^* cells (Fig. 4D). Notably, *Akt1^−/−^* cells showed much more p53 expression than *Akt1^+/+^* cells (Fig. 4D), which is consistent with our observation of more apoptotic cells in *Akt1^−/−^* cells. Interestingly, increased *Akt2* and *Akt3* expression was detected in *Akt1^−/−^* cells compared to *Akt1^+/+^* cells (Fig. 4E). Additional analysis on multiple breast tumor samples from *Akt1^−/−^* mice also showed increased expression of p53 and Cyto C compared with *Akt1^+/+^* mice (Fig.4F).

## DISCUSSION

The current studies demonstrated that miR-17/20 induces apoptosis in response to the DNA damaging agents doxorubicin and tamoxifen. MiR-17/20 transduction of MCF-7 cells induced p53 and apoptosis characterized by induction of BAX, release of cytochrome C and induction of Bcl-X(S). Using *Akt1* knockout mammary epithelial cells, and breast cancer derived cell lines from transgenic mice, we demonstrated the induction of p53 by miR-17/20 required *Akt1*. *Akt1* inhibited p53 abundance in cultured mammary epithelial cells. These studies identify a novel pathway by which the non-coding genome mediates apoptosis in response to DNA damaging agents, and thereby enhances sensitivity to drug treatment.

In the current studies, miR-17/20 enhanced tamoxifen-induced apoptosis. Addition of tamoxifen induced apoptosis with a 4-fold increase in cellular apoptosis at 24 hours. In prior studies, tamoxifen-induced apoptosis was shown to involve the estrogen receptor ERα and additional signaling kinases, including protein kinase C, calmodulin TGF-α, map kinases including JNK and p38 map kinase, and oxidative stress involving the mitochondrial permeability transition (38)(Reviewed in (39)). 10 uM tamoxifen treatment induces genotoxic stress in MCF-7 cells via a DNA damage response through free-radical production (40).

Consistent with the role of the miR-17/20 in growth inhibition, the human miR-17/20 cluster, which is located on chromosome 13q31, undergoes loss of heterozygosity in a number of malignancies, including breast cancer. In transgenic mice, miR-17/20 overexpression reduced overall tissue growth resulting in small organs (41). In breast cancer cells, the cell cycle is controlled through a cyclin D1-miR-17/20 auto-regulatory feedback loop (33). Consistent with a model in which miRNA-17 retards cellular growth in transgenic mice, these studies demonstrated miR-17/20 inhibits *cyclin D1* gene expression via a 3' UTR binding site. The cyclin D1 3' UTR is spliced in the normal population (41). The miR-17/20-mediated inhibition of breast cancer cellular proliferation via cyclin D1 repression, together with the finding that miR-17/20 inhibits breast cancer cell invasiveness (42), is consistent with a model in which miR-17/20 is a negative growth regulator in breast cancer cells. In this regard, miR-17/20 levels were reduced in highly invasive breast cancer cell lines and node-positive breast cancer specimens (42). Furthermore, cell conditioned media from miR-17/20 overexpressing MCF-7 cells inhibited the invasiveness of MDA-MB-231 cells by inhibiting secretion of several cytokines and plasminogen activators. In the current work we found the apoptosis induction by miR-17/20 in breast cancer cells. miR-17/20 overexpression induced the expression of p53, Bax, Cyto C and caspases which are key components of p53-mediated apoptosis pathway (Figure 2A and Supplementary Figure S4).

In terms of same “seed” sequence shared by miR-17 and miR-20a,1,220 conserved target genes for both miR-17 and miR-20a are predicted by TargetScan tool, in which quite a few genes are involved in cellular apoptosis regulation including caspase 7, caspase 2, BCL2-like 11, et al. Ingenuity Pathway Analysis (IPA) on the 1,220 predicted target genes revealed a cell death network (Supplemental Figure S5) and a cellular apoptosis pathway (Supplemental Figure S6).

Deletion of *Akt1* abrogated both the inhibition of cellular proliferation and the induction of apoptosis by miR-17/20. In the current studies, *Akt1* was required for miR-17/20-mediated induction of p53 abundance. These findings are consistent with prior observations that *Akt1* stabilized p53 in response to DNA damaging agents. Double-stranded DNA breaks induce cellular lesions that may lead to chromosomal rearrangements and instability. Defense mechanisms to maintain stability include cell cycle arrest, DNA repair, and apoptosis. The double-stranded DNA breaks induced by DNA damaging agents, including doxorubicin, as used in the current studies, are recognized by large phosphatidylinositol 3 protein kinases, including Ataxia Telangiectasia Mutated (ATM) and DNA-dependent Protein Kinases (DNA-PK) which sequentially implement the DNA damage response. In resting cells the levels of p53 protein are low due to the activity of E3 ubiquitin ligases (MDM2, Pirh2, and COP1). *Akt2* was required for irradiation-induced p53 stabilization (43). In mouse embryo fibroblasts and lymphoblasts, this DNA-PKc/Akt/GSK3á/Mdm2 signaling pathway prevented Mdm2-mediated p53 degradation. In the current studies, the *Akt1^−/−^* MEC cells expressed both *Akt2* and *Akt3*, indicating that *Akt1* is necessary in mammary epithelial cells for this signaling pathway.

## MATERIALS AND METHODS

### Cell culture and reagents

Murine mammary epithelial tumor cells (MMTV-ErbB2/*Akt1^+/+^* or MMTV-ErbB2/*Akt1^−/−^*) were isolated from MMTV-ErbB2 mammary gland tumors generated in bi-transgenic mice, and maintained as previously described (37). The NAFA cell line derived from the MMTV-NeuT transgenic mouse was cultured in DMEM containing penicillin and streptomycin (100 mg of each/L) and supplemented with 10% fetal bovine serum (FBS). Tamoxifen (MP Bio), doxorubicin (Sigma), were used at doses and for the times indicated in the individual figure legends.

### Retrovirus infection, small RNA/plasmid transfection

miR-17/20Retroviral production and infection methods were described in detail before (40). The ecotropic packaging vector, pSV-*ψ*^–^E-MLV which providesecotropic packaging helper function, was used with retrovirus to infect cells. F For cellular transfection with anti-miRNA (Ambion), RNAiMax (Invitrogen) was used following the manufacturer's instructions.

### Western blot analysis

Whole-cell lysates (50 μg) were separated by 10% SDS-PAGE, and the proteins were transferred to nitrocellulose membrane. The following antibodies were used for Western blotting: anti-cyclin D1 from Neomarker (Fremont, CA); anti-Bcl-Xs from Calbiochem (Darmstadt, Germany); anti-β-Tubulin (sc-9104), anti-β-actin (sc-47778), anti-p53 (sc-6243G), anti-p57 (sc-1040), anti-p27 (sc-528), anti-Cytochrome C (SC-7159), anti-Bax (sc-7480), anti-BCL-2 (sc-7382), anti PARP (sc-7150), anti-Caspase 9 (sc-8355), anti-Caspase 3 (sc-7148) were from Santa Cruz Biotechnology (Santa Crus, CA); anti-GDI from RTG Solutions (Gaithersburg, MD); anti-Akt1 (#2967 ), anti-Akt2 ( #5239 ), anti-Akt3 (#4059 ), anti-p-y-H2A ( #9718s ) were from Cell Signaling.

### Cell proliferation assays

MCF-7 Cells were infected with the pMSCV_puro_-miR-17/20 cluster or the pMSCV_puro_ empty vector. After puromycin selection, 4x10^4^ cells were seeded into each well of a 6-well plate in triplicate and cell number was counted daily for 3 to 4 days under a microscope using a hemocytometer. For the 3-(4,5-dimethylthiazol-2-yl)-2,5-diphenyltetrazolium(MTT) assay, 4x10^3^ cells/well were seeded into a 96-well plate in triplicate and cell growth was measured after 24 hours' culture.

### TUNEL assay

miR-17/20 transduced cells and control were cultured in medium containing doxorubicin (0.05μM). After 24-48 hours cells were plated into 96-well plate in triplicate. Apoptosis assays were performed using the *In Situ* Cell Death Detection Kit, TMR red (Roche Diagnostics, Mannheim, Germany) following the manufacturer's instructions.

### Annex V staining

*Akt1^+/+^* and *Akt1^−/−^* murine ErbB2 breast tumor cells were incubated with fluorochrome-conjugated Annexin V followed by Propidium Iodide staining solution treatment. Flow cytometry analysis was applied to measure apoptotic cells.

### miRNA target gene prediction and pathway analysis

The target gene prediction of hsa-miR-17 and hsa-miR-20 was performed using TargetScan Human 6.2 version (released June 2012) (http://www.targetscan.org/). The predicted target genes were further analyzed for pathway analysis and network analysis using Ingenuity Pathway Analysis (IPA).

### Statistical analysis

Data are presented as mean + SEM. The standard two-tailed student's t-test was used for analysis and P<0.05 was considered significant.

## SUPPLEMENTARY FIGURES


