# Supplementary material for: miR-17/20 sensitization of breast cancer cells to chemotherapy-induced apoptosis requires Akt1
Source: Oncotarget. 2014 Mar 4;5(4):1083–90. doi: 10.18632/oncotarget.1804 (PMC4011585; doi:10.18632/oncotarget.1804)
Supplement: Supplementary file 1 [file oncotarget-05-1083-s001.pdf]

miR-17/20 sensitization of breast cancer cells to chemotherapy-induced apoptosis requires *Akt1* – Yu et al

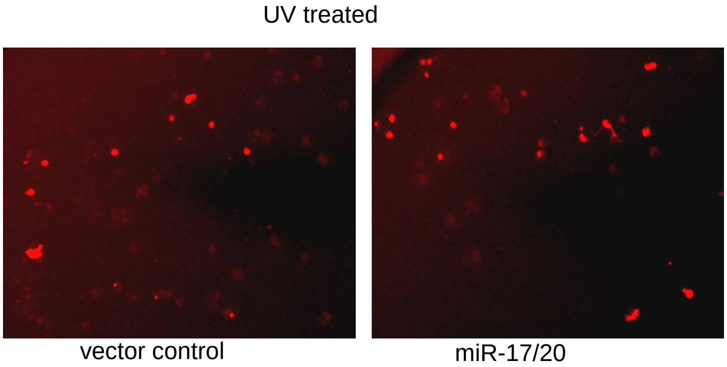

Figure S1: miR-17/20 enhancers UV-induced apoptosis. Tunnel assay of miR-17/20 transduced MCF-7 cells exposed UV radiation (20 J/m^2).

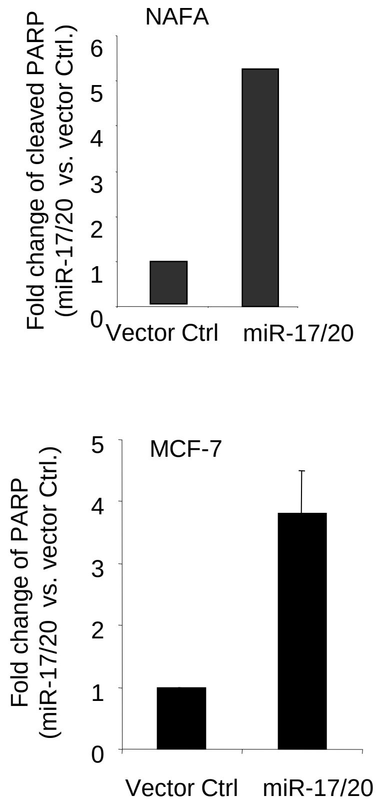

Figure S2: Densitometry analysis of the autoradiograms indicating the fold induction of PARP by miR-17/20 in NAFA and MCF-7 cells, which are shown in Figure 1G and Figure 2A by western blots.

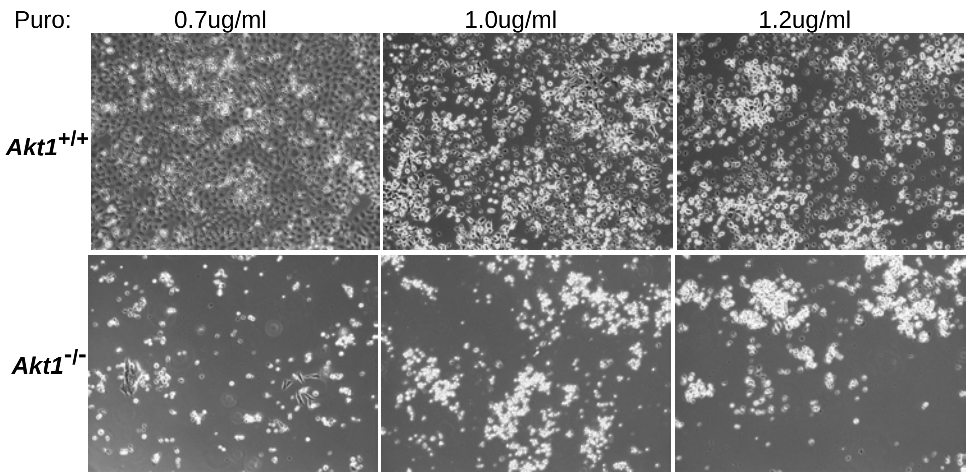

Figure S3: Increased sensitivity of *Akt1*$^{-/-}$ mammary tumor cells to genotoxic stress. Phase contrast showing the *Akt1*$^{-/-}$ mouse breast tumor cells are more sensitive to puromycin treatment (48h) compared to *Akt1*$^{+/+}$ mouse breast tumor cells.

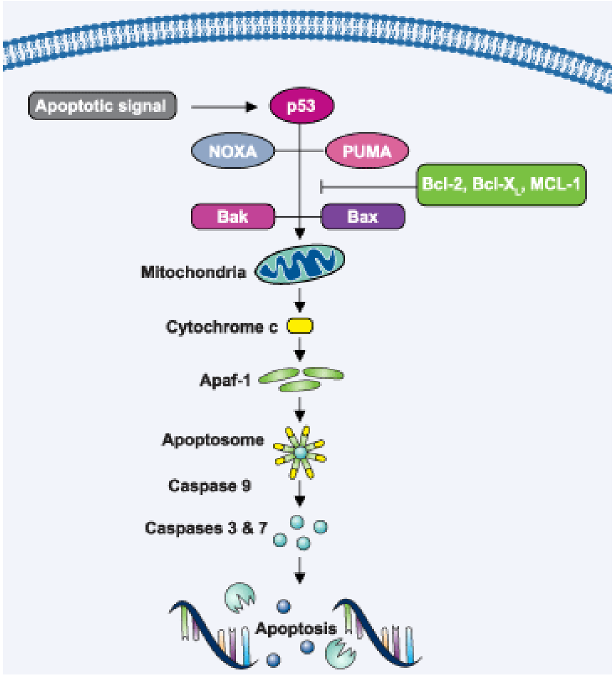

Figure S4: p53-mediated apoptosis pathway.

Cell death network*

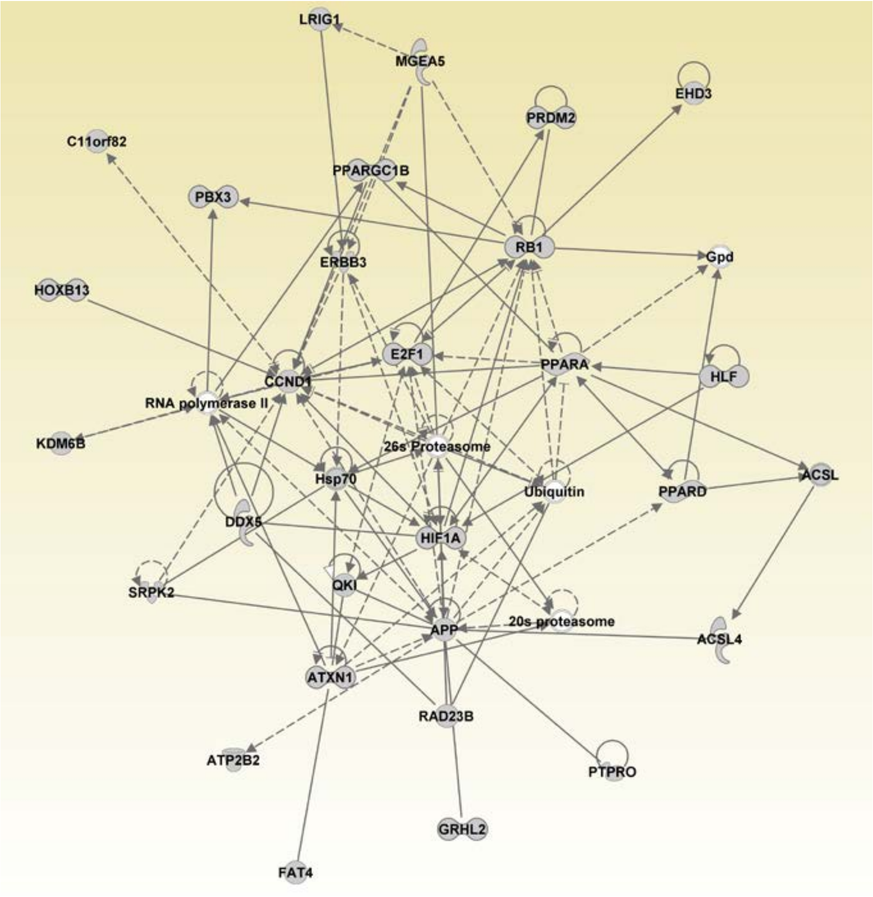

* Symbols with gray are predicted targets of miR-17/20 by TargetScan.

Figure S5: A cell death network composed of miR-17/20 target genes predicted by TargetScan.

Cellular apoptosis pathway**

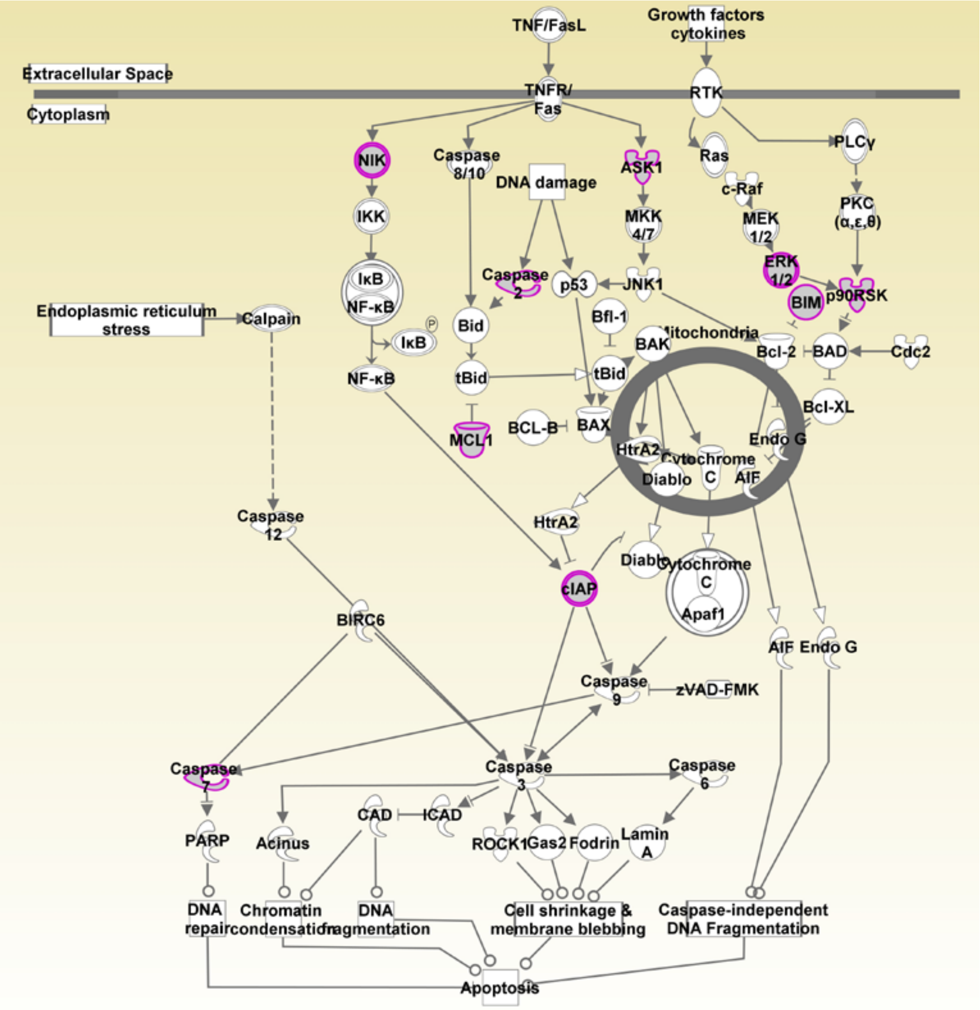

** Symbols with pink are predicted targets of miR-17/20 by TargetScan.

Figure S6: Predicted target genes of miR-17/20 involved in regulation of cellular apoptosis pathways.
